# Dietary patterns are not associated with overweight and obesity in a sample of 8900 Chinese preschool children from four cities

**DOI:** 10.1017/jns.2018.15

**Published:** 2018-09-11

**Authors:** Erigene Rutayisire, Xiaoyan Wu, Kun Huang, Shuman Tao, Yunxiao Chen, Sufang Wang, Fangbiao Tao

**Affiliations:** 1 Department of Maternal, Child & Adolescent Health, School of Public Health, Anhui Medical, University, Hefei 230032, People's Republic of China; 2 Anhui Provincial Key Laboratory of Population Health & Aristogenics, Hefei 230032, People's Republic of China; 3 Department of Public Health, School of Health Sciences, Mount Kenya University Rwanda, Kigali-Kicukiro, PO Box 5826 Kigali, Rwanda

**Keywords:** Dietary patterns, Childhood obesity, Preschool children, Factor analysis, Food frequency questionnaires

## Abstract

Globally, the prevalence of childhood obesity has substantially increased at an alarming rate. This study investigated associations between dietary patterns and overweight/obesity in 3- to 6-year-old children. Recruited children were from four prefecture-level cities in Eastern China. Childhood overweight and obesity were defined according to WHO Child Growth Standards. Individual dietary patterns were assessed by a comprehensive self-administered FFQ using thirty-five food items. Using factor analysis two dietary patterns were derived: the traditional Chinese pattern was characterised by high consumption of cereals, vegetables and fresh juices while the modern pattern was characterised by high consumption of Western fast food, Chinese fast food, sweets/sugary foods and carbonated beverages. The associations of dietary patterns with overweight/obesity were evaluated by logistic regression models. Data of 8900 preschool children from thirty-five kindergartens recruited from March to June 2015 were used in the final analysis. Adherence to the modern dietary pattern was positively associated with children's age while adherence to the traditional dietary pattern was positively associated with maternal education; these associations were statistically significant. After adjustment, we found that being in the highest tertile of any identified dietary patterns was not significantly associated with overweight and obesity. Dietary patterns are not associated with overweight/obesity in Chinese preschool children. Prospective studies are needed to establish a causal link between dietary patterns and childhood obesity.

Globally, prevalence rates of obesity and nutrition-related chronic diseases across all ages and sexes are rising^(^^1^^,^^2^^)^. Childhood overweight and obesity have become a major public health concern worldwide, as their prevalence has substantially increased at an alarming rate^(^^3^^)^. Globally, the prevalence of overweight and obesity increased from 4·2 % in 1990 to 6·7 % in 2010 among preschool children^(^^4^^)^. The prevalence of obesity among Chinese preschool children was estimated to be 10·1 % in 2014^(^^5^^)^.

The increasing rates of obesity in Chinese children may be partly explained by rapid economic development and dramatic changes in lifestyles over the past two decades^(^^6^^)^. The decreasing consumption of cereals and starchy roots, and an increasing consumption of high-energy foods have been observed among the Chinese population^(^^7^^)^. More recently, a study conducted among Chinese children and adolescents indicated that the modern dietary pattern (milk, eggs and fast food) was associated with an increased risk of obesity^(^^8^^)^. It is well known that once childhood obesity is present, it is most likely to persist into adolescence and adulthood^(^^9^^)^. Therefore, the burden of obesity is of great concern because childhood obesity leads to several immediate and long-term health problems such as insulin resistance, abnormal glucose metabolism, hypertension, dyslipidaemia, inflammation, liver disease and compromised vascular function^(^^10^^–^^14^^)^.

The effect of dietary intake on childhood obesity has received a little attention. Considering that nutrients are not eaten in isolation, analysing the effect of single nutrients or food groups on obesity ignores the inherent complexity of diet. Experts have argued that the overall pattern of dietary intake should be considered when investigating relationships between nutrition and chronic diseases such as obesity^(^^15^^,^^16^^)^. The most commonly used method of dietary pattern identification is principal component analysis, which groups correlated food items together and thereby identifies combined patterns from different types of foods. Dietary pattern analysis is population specific that may be influenced by socio-cultural factors and food availability^(^^17^^)^.

The associations between high consumption of sweet foods, snacks, fast foods and risk of overweight/obesity have previously been reported among children older than 6 years, although not all findings have been consistent^(^^8^^,^^18^^–^^20^^)^. Few studies have investigated the influence of dietary patterns on obesity risk among preschoolers. For example, a Korean study showed that high animal food consumption increased the risk of overweight among preschool children^(^^21^^)^. In China, one recent study reported the association between modern dietary patterns and risk of overweight and obesity in children and adolescents aged 7–17 years^(^^8^^)^. Based on the limited and inconsistent findings from previous studies, it is critical to understand whether dietary patterns increase the risk of obesity among preschoolers. Therefore, the present study investigated associations between dietary patterns, and overweight and obesity among children aged 3–6 years.

## Methods

### Study population

Data were collected from March to June 2015. A cluster sampling technique was used to select thirty-five kindergarten schools in four prefecture-level cities. Three prefecture-level cities (Wuhu, Tongling and Anqing) in Anhui province and one prefecture-level city (Yangzhou) in Jiangsu province were purposively selected. In Anhui province and Jiangsu province, twenty-eight and seven kindergartens were selected, respectively. In terms of socio-economic status, Jiangsu is more developed compared with Anhui province, but there is no marked socio-economic difference in the four selected small prefecture-level cities. A total of 9103 children were enrolled in the study and given the questionnaire to be completed by parents or caretaker; of them, 9006 children returned the questionnaire in the given period. Due to incomplete information, data for 106 children were excluded from the final analysis. Finally, a sample of 8900 children aged 3–6 years was used in the final analysis. The response rate of the survey was 97·7 %.

### Anthropometric measures

Data on weight and height for all children were collected in person by trained personnel at each selected kindergarten. Children were requested to wear light clothing and stand erect, barefoot, and at ease while being measured. Weight was recorded to the nearest 0·1 kg with a standardised scale and height to the nearest 0·1 cm with a portable stadiometer. Both scales and stadiometers were calibrated before use. The average of two height measures was used; in the case the two differed by greater than 0·5 cm, a third measurement was taken and the average of the two closest was used in all analyses. Overweight and obesity were defined according to WHO Child Growth Standards for age- and sex-specific cut-off points^(^^22^^,^^23^^)^. Overweight/obesity was defined as BMI of >85th percentile for age and sex. For the purpose of the study, children with BMI <85th percentile were classified in the normal weight category.

### Measures of dietary intakes

Child dietary patterns were assessed by a comprehensive and self-administered FFQ, completed by parents or caregivers. The parent or caregiver was asked to indicate his/her child's frequency of each food or food group consumed in the last 7 d prior to the survey. As there was no validated FFQ previously used to measure children's dietary patterns in China, we adapted a FFQ used to measure adolescents’ dietary patterns^(^^24^^)^. The adapted FFQ has not been validated for estimating total intakes of energy or nutrients but it was found to be appropriate for exploring dietary patterns on the basis of frequencies^(^^24^^)^. The FFQ used included thirty-five foods items mostly consumed by young children in China. The Chinese diet has a variety of foods; this make it difficult for parents/caregivers to recall portion size of foods that their children have consumed. Consequently, information on portion size was not included in the FFQ, and total energy and nutrient intakes were not estimated. The five alternative frequencies for food and drink ranged from ‘never’ to ‘three or more times’ per d. Each option was scored as follows: ‘never eat’ = 1; ‘one time’ = 2; ‘two times = 3’; ‘three times = 4’; and ‘more than three times’ = 5.

### Other relevant variables

Participants’ characteristics were self-reported by parents at home and the completed questionnaire was returned to school within a 2-week period. Asked information included maternal age in years; maternal education and maternal smoking. Child-related factors included age, sex (male/female) and having a sibling (yes/no). Paternal factors included age, education level and smoking status. Family monthly income was self-reported by parents through the same questionnaire, based on reported monthly income; we classified subjective family income in the following ways: low income; middle income; and high income.

Parental BMI was calculated as weight in kilograms divided by height in metres, squared (kg/m^2^) and was classified according to the WHO reference: normal weight (BMI less than 25·0 kg/m^2^), overweight (BMI 25·0–29·9 kg/m^2^) and obese (BMI 30·0 kg/m^2^ or more)^(^^25^^)^. A combined variable for parents’ weight status was constructed by combining mother's and father's BMI variables and was coded as: both parents having normal weight; only one parent has normal weight; and both parents overweight/obese.

### Ethics of human subjects’ participation

Approval for the study was obtained from the ethical committee of Anhui Medical University. Written informed consent was obtained from the parents of all children who participated in the study.

### Statistical analysis

Factor analysis, a principal component analysis of food frequency consumption data, was conducted based on the thirty-five food groups to derive dietary patterns. The factors were rotated by varimax rotation, and two dimensions were extracted from the rotated component matrix. Both dimensions had an eigenvalue >1. Factor loadings indicated the strength and direction of the association between the patterns and food groups. Food groups with positive loadings contribute to the dietary pattern, whereas those with negative loadings are inversely associated with the dietary pattern. Food items were retained in a factor if they had an absolute correlation >0·3 with that factor. When a food group loaded high (>0·3) in more than one factor labelling, the factor with the highest loading was considered for factor labelling. Factor scores were calculated by the multiple regression approach, and each individual received a factor score for each dietary pattern. These scores indicated the degree to which each subject's diet corresponded to the identified pattern.

We used the χ^2^ test for univariable analysis to establish the relationship between participants’ characteristics and preschoolers’ BMI. The variables with significant association from univariate analysis were used in model 1 and model 2 as potential confounders on the associations between dietary patterns and overweight and obesity. Multiple linear regression analyses were used to examine the correlation between dietary patterns and participants’ characteristics. The dependent variables in regression models were factor scores of the identified patterns, whereas independent variables included child's, maternal and paternal characteristics as well as monthly family income.

The associations of dietary patterns with overweight and obesity were evaluated by means of multiple logistic regression models, with tertiles of dietary patterns’ scores as independent variables and the overweight and obesity indices as outcome variables. OR and 95 % CI were estimated. In model 1, we adjusted for sex (male/female), age (continuous), having a sibling (yes/no) while in model 2, we adjusted for maternal education, paternal smoking status, parental BMI (parents with normal weight, one parent with normal weight, both parents overweight or obese) plus variables adjusted in model 1. *P* values less than 0·05 were regarded as statistically significant. All statistical analyses were performed using SPSS, version 10.

## Results

Two dietary patterns were derived from thirty-five food groups in the FFQ using principal component analysis: modern and traditional. The two dietary patterns explained 37·8 % of the total variance in dietary intake. The traditional Chinese pattern was characterised by wheat and other cereals, tubers, legumes, fruits, vegetables, fresh juices, eggs, low-fat dairy products, poultry and fish. The modern pattern was characterised by Western fast food, Chinese fast food, preserved food, fried vegetables, fried meats, sweet course, sugary foods, chocolates/ice cream, carbonated beverages, flavoured milk drinks and synthesised fruit/vegetable juice. For each pattern, participants were grouped into tertiles of pattern scores. Factor loadings and the variance explained by each factor are shown in Table 1.

**Table 1.** Factor-loading matrix for the two dietary patterns and their food or food groups identified in 8900 Chinese preschool children*

	Dietary pattern	
Food or food groups	Modern	Traditional
Wheat (steamed bread, bread)	–	0·456
Other cereals (maize, sorghum, oats)	0·319	0·515
Tubers	0·329	0·518
Legumes	–	0·563
Fruits	–	0·464
Fresh green leafy vegetables	–	0·571
Fresh red or yellow vegetables	–	0·657
Other vegetables	–	0·628
Fresh fruit and fresh vegetable juice	0·308	0·483
Red meat (pork, beef, mutton, rabbit)	–	0·536
Poultry (chicken, duck, goose)	–	0·613
Fish and other aquatic products	–	0·563
Processed meat	0·421	0·464
Animal blood products	0·457	0·428
Organ meat	0·397	0·413
Eggs	–	0·446
Low-fat dairy products	–	0·394
High-lipid dairy products	0·455	0·305
Yogurt	0·311	0·370
Soya milk	0·441	0·387
Preserved fruits	0·575	0·377
Nuts	0·510	0·418
Pancake	0·668	–
Preserved food	0·627	–
Chinese fast food	0·684	–
Fried vegetables	0·724	–
Fried meats	0·723	–
Western fast food (sandwiches, hamburgers)	0·698	–
Sweet course (cakes, cookies)	0·507	–
Sugary foods (candy, white sugar, brown sugar)	0·562	–
Chocolate/ice cream	0·626	–
Puffed food	0·700	–
Carbonated beverages	0·689	–
Synthesised fruit/vegetable juice	0·697	–
Flavoured milk drink (milk beverage)	0·682	–
Percentage variance explained	22·2	15·5

* Absolute values <0·30 are not presented in the table for simplicity.

Participants’ characteristics by child BMI category are presented in Table 2. A total of 2326 children (26·1 %) were overweight/obese; of them 62·9 % were male. Sex, age, having a sibling, maternal education, paternal education, paternal smoking and parental BMI were significantly associated with child BMI. Maternal age, maternal smoking, father's age and family income were not associated with child BMI.

**Table 2.** Participant characteristics by child BMI category (Numbers of participants and percentages; mean values and standard deviations)

	Normal		Overweight/obesity		
	*n*	%	*n*	%	*P*
*n*	6574	73·9	2326	26·1	
Sex					
Male	3248	49·4	1462	62·9	<0·001
Female	3326	50·6	864	37·1	
Age (years)					<0·001
Mean	4·33		4·46		
sd	0·99		1·01		
Having a sibling					
Yes	1372	20·9	421	18·1	0·004
No	5202	79·1	1905	81·9	
Mother's age (years)					
<30	1824	27·7	625	26·9	
30–35	3710	56·4	1327	57·1	0·551
36–40	826	12·6	286	12·3	
41 or older	214	3·3	88	3·8	
Maternal education					
<High school	1102	16·8	315	13·5	
High school	1571	23·9	569	24·5	0·001
College and above	3901	59·3	1442	62·0	
Maternal smoking					
Never	6527	99·3	2317	99·6	
One or fewer cigarettes per d	16	0·2	–		
1–5 cigarettes per d	21	0·3	5	0·2	0·095
6 or more cigarettes per d	10	0·2	4	0·2	
Father's age (years)					
<30	786	12·0	270	11·6	
30–35	3561	54·2	1314	56·5	0·270
36–40	1519	23·1	509	21·9	
41 or older	708	10·8	233	10·0	
Paternal education					
<High school	2309	35·1	749	32·2	
High school	1722	26·2	646	27·8	0·036
College and above	2543	38·7	931	40·0	
Paternal smoking					
Never	3074	46·8	996	42·8	
One or fewer cigarettes per d	342	5·2	148	6·4	0·004
1–5 cigarettes per d	1273	19·4	488	21·0	
6 or more cigarettes per d	1885	28·7	694	29·8	
Parental BMI					
Both parents with normal weight	4054	61·7	1099	57·2	
One parent overweight or obese	2309	35·1	1058	45·5	<0·001
Both parents overweight or obese	211	3·2	169	7·3	
Family income					
Lower	2868	43·6	1058	45·5	
Middle	2938	44·7	1030	44·3	0·100
Higher	768	11·7	238	10·2	

Multivariate linear regression models were used to examine the independent associations between the sociodemographic characteristics with the scores of the identified dietary patterns. Table 3 represents the regression coefficients and the corresponding 95 % CI describing these associations. Adherence to the modern dietary pattern was positively associated with child age (β 0·024; 95 % CI 0·004, 0·045) and having a sibling (β 0·093; 95 % CI 0·043, 0·145). This pattern was negatively associated with maternal age (β −0·016; 95 % CI −0·021, −0·011), maternal education (β −0·156; 95 % CI −0·184, −0·129), paternal age (β −0·008; 95 % CI −0·013, −0·004), paternal education (β −0·123; 95 % CI −0·147, −0·099) and paternal smoking (β −0·032; 95 % CI −0·048, −0·016). Moreover, these associations were statistically significant.

**Table 3.** Association of sociodemographic characteristics with dietary patterns in Chinese preschool children from four cities in East China (β-Coefficients and 95 % confidence intervals; *n* 8900)

	Dietary patterns			
	Traditional Chinese		Modern	
	*β*	95 % CI	*β*	95 % CI
Age (years)	0·020	0·000, 0·041	0·024*	0·004, 0·045
Females *v.* males	0·021	−0·021, 0·063	0·000	−0·043, 0·041
Having *v.* not having a sibling	−0·031	−0·082, 0·021	0·093*	0·043, 0·145
Maternal age	−0·004	−0·010, 0·001	−0·016*	−0·021, −0·011
Maternal education	0·114*	0·086, 0·142	−0·156*	−0·184, −0·129
Maternal smoking	0·035	−0·099, 0·168	−0·014	−0·120, 0·148
Paternal age	−0·006*	−0·010, −0·001	−0·008*	−0·013, −0·004
Paternal education	0·080*	0·056, 0·104	−0·123*	−0·147, −0·099
Paternal smoking	0·032*	0·015, 0·048	−0·032*	−0·048, −0·016
Family income	−0·077*	−0·108, −0·046	0·019	−0·012, 0·051
Parental BMI	−0·004	−0·040, 0·032	0·005	−0·031, 0·041

* *P* < 0·05.

On the other hand, adherence to the traditional dietary pattern was positively associated with maternal education (β 0·114; 95 % CI 0·086, 0·142), paternal education (β 0·080; 95 % CI 0·056, 0·104) and paternal smoking (β 0·032; 95 % CI 0·015, 0·048). This pattern was negatively associated with paternal age (β −0·006; 95 % CI −0·010, −0·001) and family income (β −0·077; 95 % CI −0·108, −0·046). Furthermore, these associations were statistically significant.

Logistic regression analyses on the associations between dietary patterns and overweight/obesity are shown in Table 4. In model 1, compared with children in the lowest tertile of a dietary pattern's score, being in the highest tertile of the modern pattern was not significantly associated with childhood overweight and obesity (OR 0·916; 95 % CI 0·816, 1·030). After controlling for maternal education, paternal smoking and parental smoking in addition to variables adjusted in model 1 in this pattern, however, the results remained not statistically significant at *P* < 0·05. In both models, there were no significant associations between the traditional dietary pattern and overweight/obesity among the children in the highest tertile of the traditional dietary pattern compared with those in the lowest tertile.

**Table 4.** Association of dietary patterns with childhood overweight and obesity (Odds ratios and 95 % confidence intervals)

	Dietary patterns					
	Traditional Chinese			Modern		
	OR	95 % CI	*P* for trend	OR	95 % CI	*P* for trend
Model 1*						
*T*_1_ = 2966	1·0			1·0		
*T*_2_ = 2968	1·057	0·941, 1·189	0·643	0·904	0·805, 1·016	0·183
*T*_3_ = 2966	1·024	0·911, 1·152		0·916	0·816, 1·030	
Model 2†						
*T*_1_ = 2966	1·0			1·0		
*T*_2_ = 2968	1·058	0·940, 1·191	0·642	0·902	0·801, 1·015	0·199
*T*_3_ = 2966	1·021	0·906, 1·151		0·924	0·820, 1·041	

*T*_1_, lowest tertile of dietary pattern score; *T*_2_, intermediate tertile of dietary pattern score; *T*_3_, highest tertile of dietary pattern score.

* Model 1: adjusted for sex (male/female), age (continuous), having a sibling (yes/no).

† Model 2: maternal education, paternal smoking status, parental BMI (parents with normal weight, one parent with normal weight, both parents overweight or obese).

## Discussion

The purpose of the present study was to examine the associations between dietary patterns and childhood overweight and obesity. No significant association was found between any identified dietary patterns and overweight/obesity. In the present study, we identified two dietary patterns: the modern dietary pattern and the traditional Chinese pattern. The modern pattern was characterised mainly by high intakes of Western fast food, Chinese fast food, fried meats, preserved food, sweets, sugary foods, chocolate and ice cream, flavoured milk and carbonated beverages. The traditional Chinese pattern reflected high intakes of cereals, tubers, legumes, fruits, vegetables, poultry, fish, eggs and low-fat dairy products. These two patterns identified in our study are almost similar to those extracted among young Chinese children and adolescents^(^^8^^,^^24^^)^. For example, Zhang *et al*.^(^^8^^)^ identified the modern pattern characterised by high intakes of fast foods and milk.

In the present study, there were differences regarding the children's adherence to the pattern according to their sociodemographic characteristics. Specifically, we observed that adherence to the traditional Chinese pattern was associated with high parental education level. These results are in agreement with those reported among European children and adolescents^(^^18^^,^^26^^,^^27^^)^. Our findings highlight the influence of parental education in dietary intake and food preferences among preschool children. Unexpectedly, we found that being in the top class of preschool as well as having a sibling was positively associated with adherence to the modern dietary pattern. These findings may be partly explained by that food preference for children in the transition from kindergarten to primary school may be influenced by environments, availability, younger siblings, and thus parents may be less able to exert control over their child's food consumption. A recent study reported that younger siblings received more encouragement to eat from their older siblings^(^^28^^)^; this may include high energy-dense foods that characterise the modern pattern in the present study.

No significant associations were found of modern dietary patterns or the traditional Chinese pattern with overweight/obesity among preschool children. In line with our findings, a study on Australian adolescents reported no associations between any of the identified dietary patterns and BMI^(^^29^^)^. No association was found between children in the highest tertile of the modern pattern and the risk of overweight/obesity. In contrast, a recent study conducted among Chinese children and adolescents aged 7–17 years showed that subjects in the highest quartiles of the modern dietary pattern had increased risk of obesity^(^^8^^)^. Another study in Lebanon reported that adolescents in the highest tertile of the Western dietary pattern had higher risk of overweight compared with adolescents in the lowest tertile^(^^18^^)^. The modern/Western pattern in these studies was mainly characterised by increased consumption of milk, fast foods, beverages, sugary foods and poultry, which are similar to the foods reflected in the modern pattern of our study. A Korean study conducted among preschool children revealed that children in the highest quartile of animal foods had a 77 % increased risk of overweight compared with children in the lowest quartile of that pattern^(^^21^^)^. Generally, it is difficult to compare previous findings with the present results due to the age that outcome was assessed in previous studies, and dietary patterns’ characteristics. Additionally, confounding factors could also play an intermediate role. For example, a study of Australian children aged 12–18 years suggested that the association between dietary patterns and BMI could be due to controlled confounding^(^^29^^)^.

In line with our findings, several studies reported no significant association between BMI and the southern Chinese traditional pattern^(^^8^^)^, Korean healthy pattern^(^^21^^)^ and Lebanese traditional pattern^(^^18^^)^. However, most of these studies were conducted among older children and adolescents; very few reported the influence of the traditional pattern on overweight and obesity among preschool children. Indeed, the traditional pattern of this study was mainly characterised by increased consumption of cereals, legumes, fruits, fresh vegetables, fresh juice and low-fat dairy products which were also considered as healthy foods in previous studies.

The risk of overweight and obesity in preschool children might be linked to genetic factors^(^^30^^)^, screen time^(^^31^^)^ and other environmental factors rather than dietary components alone. Farooqi & O'Rahilly^(^^32^^)^ estimated the heritability of BMI at around 40 to 70 %. This evidence shows that the contribution of genetic factors stems from the genes of parents on the development of childhood overweight and obesity during the preschool period. A better understanding of the lifestyle, family and environmental factors linked with childhood obesity may pave avenues toward the prevention of obesity and related diseases.

The two identified dietary patterns have no significant effect on overweight and obesity in 3- to 6-year-old children. However, given the high prevalence of overweight and obesity reported in our study, the exact mechanism that led to childhood obesity needs to be elucidated. We speculate that parental BMI and other environmental factors contribute to the prevalence of childhood obesity. Therefore, parents may play an important role in the reduction in the current childhood obesity epidemic. We therefore suggest that parents’ opportunity to influence positively their children's activity and diet must be used to decrease overweight and obesity rates among preschool children. To our knowledge this is the first study conducted in two provinces in China that characterised dietary patterns in relation to children's BMI in Chinese preschoolers.

The main strength of this study is the large representative sample of 3- to 6-year-old preschool children, data from different cities as well as different kindergarten schools. Compared with an analysis based on individual foods or nutrients, the use of dietary patterns may be more informative for investigating the implication of the combinations of foods in childhood obesity as well as the potentially synergistic effects of foods and nutrients.

Several limitations should be noted, including the inability to adjust for potential confounders such as parental dietary pattern, children's screen time and physical activity. The present study was a cross-sectional analysis, and thus causal inference cannot be made. Longitudinal studies are required to further investigate associations between dietary patterns and childhood obesity. Recall bias cannot be ruled out as many variables were self-reported by parents. Another potential limitation of this study is the lack of portion size data on the FFQ; thus, energy intake was not estimated. However, previous studies have suggested that energy adjustment of food intake in the dietary patterns derived by factor analysis is not necessary as the majority of variation in food intakes may be captured by frequency of consumption^(^^33^^,^^34^^)^.

### Conclusion

Dietary patterns were not statistically associated with overweight and obesity. Being in the highest tertile of adherence to the modern pattern was not significantly associated with overweight and obesity. Prospective studies are needed to establish a causal link between dietary pattern and childhood obesity. Additional confounders such as parental dietary pattern and child's physical activity should be considered.

## References

[1] Popkin BM (2010) Recent dynamics suggest selected countries catching up to US obesity. Am J Clin Nutr 91, 284S–288S. 19906804 10.3945/ajcn.2009.28473C PMC2793114

[2] Popkin BM, Adair LS & Ng SW (2012) Global nutrition transition and the pandemic of obesity in developing countries. Nutr Rev 70, 3–21. 22221213 10.1111/j.1753-4887.2011.00456.x PMC3257829

[3] World Health Organization (2016) Report of the Commission on Ending Childhood Obesity. Geneva: WHO. http://apps.who.int/iris/bitstream/handle/10665/204176/9789241510066_eng.pdf;jsessionid=2EAC8B81F0C80876FBB1EFD2AC683F14?sequence=1 (accessed August 2018).

[4] de Onis M, Blossner M & Borghi E (2010) Global prevalence and trends of overweight and obesity among preschool children. Am J Clin Nutr 92, 1257–1264. 20861173 10.3945/ajcn.2010.29786

[5] Xiao Y, Qiao Y, Pan L, (2015) Trends in the prevalence of overweight and obesity among Chinese preschool children from 2006 to 2014. PLOS ONE 10, e0134466. 26267264 10.1371/journal.pone.0134466 PMC4534378

[6] Ji CY & Cheng TO (2009) Epidemic increase in overweight and obesity in Chinese children from 1985 to 2005. Int J Cardiol 132, 1–10. 18835050 10.1016/j.ijcard.2008.07.003

[7] Zhai F, Wang H, Du S, (2009) Prospective study on nutrition transition in China. Nutr Rev 67, Suppl. 1, S56–S61. 19453679 10.1111/j.1753-4887.2009.00160.x

[8] Zhang J, Wang H, Wang Y, (2015) Dietary patterns and their associations with childhood obesity in China. Br J Nutr 113, 1978–1984. 25944159 10.1017/S0007114515001154 PMC4594803

[9] Guo SS, Wu W, Chumlea WC, (2002) Predicting overweight and obesity in adulthood from body mass index values in childhood and adolescence. Am J Clin Nutr 76, 653–658. 12198014 10.1093/ajcn/76.3.653

[10] Abdulle A, Al-Junaibi A & Nagelkerke N (2014) High blood pressure and its association with body weight among children and adolescents in the United Arab Emirates. PLOS ONE 9, e85129. 24465493 10.1371/journal.pone.0085129 PMC3896369

[11] Zhang CX, Tse LA, Deng XQ, (2008) Cardiovascular risk factors in overweight and obese Chinese children: a comparison of weight-for-height index and BMI as the screening criterion. Eur J Nutr 47, 244–250. 18587680 10.1007/s00394-008-0718-7

[12] Mansour M, Nassef YE, Shady MA, (2016) Metabolic syndrome and cardiovascular risk factors in obese adolescent. Open Access Maced J Med Sci 4, 118–121. 27275343 10.3889/oamjms.2016.037 PMC4884230

[13] Valerio G, Licenziati MR, Manco M, (2014) [Health consequences of obesity in children and adolescents]. Minerva Pediatr 66, 381–414. 25253187

[14] Franks PW, Hanson RL, Knowler WC, (2010) Childhood obesity, other cardiovascular risk factors, and premature death. N Engl J Med 362, 485–493. 20147714 10.1056/NEJMoa0904130 PMC2958822

[15] Tucker KL (2010) Dietary patterns, approaches, and multicultural perspective. Appl Physiol Nutr Metab 35, 211–218. 20383235 10.1139/H10-010

[16] Jacques PF & Tucker KL (2001) Are dietary patterns useful for understanding the role of diet in chronic disease? Am J Clin Nutr 73, 1–2. 11124739 10.1093/ajcn/73.1.1

[17] Balder HF, Virtanen M, Brants HA, (2003) Common and country-specific dietary patterns in four European cohort studies. J Nutr 133, 4246–4251. 14652380 10.1093/jn/133.12.4246

[18] Naja F, Hwalla N, Itani L, (2015) A Western dietary pattern is associated with overweight and obesity in a national sample of Lebanese adolescents (13–19 years): a cross-sectional study. Br J Nutr 114, 1909–1919. 26431469 10.1017/S0007114515003657 PMC4635384

[19] Pérez-Rodrigo C, Gil A, González-Gross M, (2016) Clustering of dietary patterns, lifestyles, and overweight among Spanish children and adolescents in the ANIBES Study. Nutrients 8, 11. 10.3390/nu8010011 PMC4728625 26729155

[20] Oellingrath IM, Svendsen MV & Brantsaeter AL (2010) Eating patterns and overweight in 9- to 10-year-old children in Telemark County, Norway: a cross-sectional study. Eur J Clin Nutr 64, 1272–1279. 20717128 10.1038/ejcn.2010.152 PMC3002052

[21] Shin KO, Oh SY & Park HS (2007) Empirically derived major dietary patterns and their associations with overweight in Korean preschool children. Br J Nutr 98, 416–421. 17433127 10.1017/S0007114507720226

[22] World Health Organization (2006) WHO Child Growth Standards: methods and development. http://wdelword_wwwhoint/nutrition/publications/childgrowthstandards_technical_report_1/en/ (accessed October 2015).

[23] de Onis M, Onyango AW, Borghi E, (2007) Development of a WHO growth reference for school-aged children and adolescents. Bull World Health Organ 85, 660–667. 18026621 10.2471/BLT.07.043497 PMC2636412

[24] Lu Q, Tao F, Hou F, (2016) Emotion regulation, emotional eating and the energy-rich dietary pattern. A population-based study in Chinese adolescents. Appetite 99, 149–156. 26792769 10.1016/j.appet.2016.01.011

[25] World Health Organization (2000) *Obesity: Preventing and Management the Global Epidemic. Report of a WHO Consultation. WHO Technical Report Series* no. 894. Geneva: WHO. 11234459

[26] Emmett PM & Jones LR (2015) Diet, growth, and obesity development throughout childhood in the Avon Longitudinal Study of Parents and Children. Nutr Rev 73, 175–206. 10.1093/nutrit/nuv054 PMC4586450 26395342

[27] Aranceta J, Perez-Rodrigo C, Ribas L, (2003) Sociodemographic and lifestyle determinants of food patterns in Spanish children and adolescents: the enKid study. Eur J Clin Nutr 57, S40–S44. 12947451 10.1038/sj.ejcn.1601813

[28] Mosli RH, Miller AL, Kaciroti N, (2015) Mealtime behavior among siblings and body mass index of 4–8 year olds: a videotaped observational study. Int J Behav Nutr Phys Act 12, 94. 26169374 10.1186/s12966-015-0256-7 PMC4501061

[29] McNaughton SA, Ball K, Mishra GD, (2008) Dietary patterns of adolescents and risk of obesity and hypertension. J Nutr 138, 364–370. 18203905 10.1093/jn/138.2.364

[30] Kostovski M, Tasic V, Laban N, (2017) Obesity in childhood and adolescence, genetic factors. Pril *(*Makedon Akad Nauk Umet Odd Med Nauki*)* 38, 121–133. 29668472 10.2478/prilozi-2018-0013

[31] Andersen RE, Crespo CJ, Bartlett SJ, (1998) Relationship of physical activity and television watching with body weight and level of fatness among children: results from the Third National Health and Nutrition Examination Survey. JAMA 279, 938–942. 9544768 10.1001/jama.279.12.938

[32] Farooqi IS & O'Rahilly S (2005) New advances in the genetics of early onset obesity. Int J Obes *(*Lond*)* 29, 1149–1152. 16155585 10.1038/sj.ijo.0803056

[33] Northstone K, Ness AR, Emmett PM, (2008) Adjusting for energy intake in dietary pattern investigations using principal components analysis. Eur J Clin Nutr 62, 931–938. 17522611 10.1038/sj.ejcn.1602789 PMC2492394

[34] Noethlings U, Hoffmann K, Bergmann MM, (2003) Portion size adds limited information on variance in food intake of participants in the EPIC-Potsdam study. J Nutr 133, 510–515. 12566492 10.1093/jn/133.2.510

